# Involvement of Cyr61 in growth, migration, and metastasis of prostate cancer cells

**DOI:** 10.1038/sj.bjc.6604712

**Published:** 2008-10-21

**Authors:** Z-J Sun, Y Wang, Z Cai, P-P Chen, X-J Tong, D Xie

**Affiliations:** 1 Institute for Nutritional Sciences, Shanghai Institutes for Biological Sciences, Chinese Academy of Sciences, Shanghai 200031, China; 2 Graduate School of the Chinese Academy of Sciences, Shanghai 200031, China; 3 College of Public Health, Zhengzhou University, Zhengzhou 450001, China; 4 College of Life Sciences, Peking University, Beijing 100871, China

**Keywords:** PCa, Du145, Cyr61, Rac1

## Abstract

Cyr61 has been reported to participate in the development and progression of various cancers; however, its role in prostate cancer (PCa) still remains poorly understood. In this study, we explored the function of Cyr61 in a series of malignant PCa cell lines, including LnCap, Du145, and PC3. 3-(4,5-Dimethylthiazol-2-yl)-2,5-diphenyltetrazolium bromide (MTT) and crystal violet assays demonstrated that Cyr61 was essential for the proliferation of PCa cells. Soft agar assay and xenograft analysis showed that downregulation of Cyr61 suppressed the tumorigenicity of Du145 cells both *in vitro* and *in vivo*. Either silencing the cellular Cyr61 by RNA interference or neutralising the endogenous Cyr61 by antibody inhibited the migration of Du145 cells. In contrast, purified protein of Cyr61 promoted the migration of LnCap cells in a dose-dependent manner. These results suggested that Cyr61 was involved in the migration of PCa cells. We also observed the accumulation of mature focal adhesion complexes associated with the impaired migration through Cyr61 downregulation. Also, further studies showed that Cyr61 regulated the level of activated Rac1 as well as its downstream targets, including phosphorylated JNK, E-cadherin, and p27^kip1^, which are key molecules involved in cell growth, migration, and invasion. The *in vivo* mouse tail vein injection experiment revealed that Cyr61 affected the metastatic capacity of Du145 cells, suggesting that Cyr61 was required for prostate tumour metastasis. Altogether, our results demonstrated that Cyr61 played an important role in the tumorigenicity and metastasis of PCa cells, which will benefit the development of therapeutic strategy for PCas.

Prostate cancer (PCa) is one of the main diseases influencing men today, ranked the third most common cancer worldwide (Ziober *et al*, 2001; Sim and Cheng, 2005). Though the molecular mechanisms underlying remain poorly understood, accumulating evidence indicates the involvement of various growth factors in the development and progression of PCa (Ware, 1998; Melton *et al*, 2007), such as insulin-like growth factor (Gennigens *et al*, 2006), vascular endothelial growth factor (Delongchamps *et al*, 2006), transforming growth factor-*β* (Zhu and Kyprianou, 2005), epidermal growth factor (Spiegel and Milstien, 2005), and hepatocyte growth factor (Hurle *et al*, 2005).

CCN growth factor family is named after the three first identified members – Cyr61 (cysteine-rich protein 61, CCN1), CTGF (connective tissue growth factor, CCN2), and Nov (nephroblastoma overexpressed, CCN3) (Bork, 1993). So far, this family has included six members: Cyr61, CTGF, Nov, WISP-1 (Wnt-1-induced secreted protein 1) (CCN4), WISP-2 (CCN5), and WISP-3 (CCN6) (Brigstock, 2003; Perbal, 2004). All CCN molecules share four conserved structural modules with sequence homologies similar to cysteine knot, thrombospondin, von Willebrand factor, and insulin-like growth factor-binding protein, respectively (Bork, 1993). Earlier studies have reported that CTGF and Nov promoted tumorigenesis of PCa (Maillard *et al*, 2001; Yang *et al*, 2005), whereas the expression of Cyr61 was downregulated in PCa (Pilarsky *et al*, 1998).

Cyr61 was the first cloned member of the CCN family and reported to mediate a variety of cellular processes, including cell adhesion, stimulation of chemostasis, enhancement of growth factor-induced DNA synthesis, cell survival, and angiogenesis (Grzeszkiewicz *et al*, 2002; Lin *et al*, 2004). Moreover, Cyr61 has been reported to be involved in the development of several kinds of tumours (Bleau *et al*, 2005). Overexpressed Cyr61 could stimulate the progression of breast cancers (Xie *et al*, 2001a, 2001b). A gastric adenocarcinoma cell line became more tumorigenic when the cells were genetically engineered to express high levels of Cyr61 (Babic *et al*, 1998). High expression level of Cyr61 was reported in rhabdomyosarcomas, malignant melanomas, colon adenocarcinomas, and bladder papillomas (Genini *et al*, 1996; Babic *et al*, 1998). Cyr61 also exhibited high levels in malignant gliomas and enhanced the tumorigenicity through the integrin-linked kinase signalling pathway (Xie *et al*, 2004). Upregulation of Cyr61 expression was recently identified in peritoneal metastases from human pancreatic cancer (Holloway *et al*, 2005). Paradoxically, Cyr61 was downregulated in lung cancers, and forced expression of Cyr61 inhibits tumorigenicity of lung cancer cells (Tong *et al*, 2001, 2004). Cyr61 was also reported to inhibit the growth of endometrial cancer (Chien *et al*, 2004) and leiomyomas (Sampath *et al*, 2001).

Although it has been reported that Cyr61 was upregulated and required for prostatic cell proliferation in benign prostatic hyperplasia (Sakamoto *et al*, 2004), there are rare reports on the role of Cyr61 in malignant PCa cells. In this study, therefore, we investigated the functions of Cyr61 in Du145, a high-grade metastatic PCa cell line (Stone *et al*, 1978), and found that Cyr61 played an essential role in the proliferation, migration, and metastasis of this PCa cells both *in vitro* and *in vivo*.

## Materials and methods

### Prostate cancer tissue samples and cell lines

Twenty pairs of primary PCa samples and their corresponding normal tissues were obtained from PCa patients treated at the First Affiliated Hospital of Zhengzhou University (Henan, China) from 2002 to 2005 after their written informed consent, and none of the patients received any neoadjuvant therapy. All specimens were frozen at once in liquid nitrogen after surgical excision and stored at −80°C until use. Our study was approved by the Institutional Review Board of the Institute for Nutritional Sciences, Chinese Academy of Sciences. PC3, Du145, LnCap, and 22RV1 cells were purchased from the American Type Culture Collection (Manassas, VA, USA) and cultured in RPMI-1640, supplemented with 10% foetal bovine serum, 10 U ml^−1^ penicillin, and 10 U ml^−1^ streptomycin, at 37°C in a humidified atmosphere containing 5% CO_2_.

### Reagents

Rabbit anti-human Cyr61 polyclonal antibody was purchased from Santa Cruz Biotechnology (SC-13100(H-78); Santa Cruz, CA, USA); anti-focal adhesion kinase (anti-FAK, clone 77), anti-Rac1 (clone 102), anti-paxillin (clone 68), FAK (pY397), Paxillin (pY118), anti-phosphorylated JNK (pT183/pY185, clone 41), and pan-p-JNK (clone 37) monoclonal antibodies were from BD Transduction Laboratories (San Diego, CA, USA); Rac1-specific inhibitor NSC23766 was purchased from EMD Biosciences (Darmstadt, Germany); Lipofectamine 2000 was from Invitrogen (Carlsbad, CA, USA); 3-(4,5-Dimethylthiazol-2-yl)-2,5-diphenyltetrazolium bromide (MTT) was from Roche Molecular Biochemicals (Basel, Switzerland); and D-luciferin was from Biosynth International Inc.(Naperville, IL, USA).

### RNA interference of Cyr61 and rescue experiments

**Figure 1:**
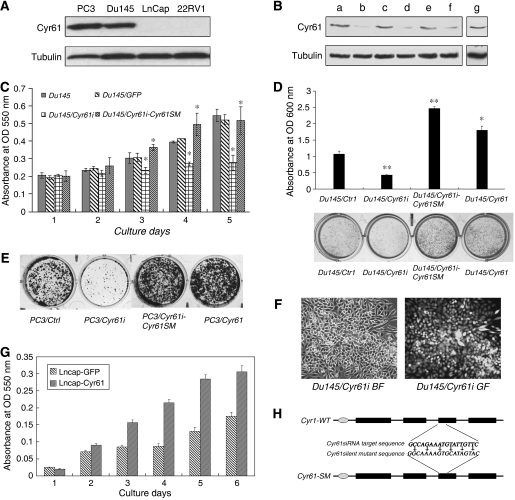
Silencing of Cyr61 inhibited the proliferation of Du145 cells. (**A**) The protein level of Cyr61 in four PCa cell lines. (**B**) Du145 cells were infected with lentivirus either expressing Cyr61 siRNA (Du145/Cyr61i) or the control siRNA (Du145/Ctrli), which processed the same A/T composition but different sequences, and not matched with any genes. Three sequences of either Cyr61 siRNA or control siRNA were designed. Lane a: Du145/Ctrli #1 cells; lane b: Du145/Cyr61i #1 cells; lane c: Du145/Ctrli #2 cells; lane d: Du145/Cyr61i #2 cells; lane e: Du145/Ctrli #3 cells; lane f: Du145/Cyr61i #3 cells; and lane g: wild-type Du145 (Du145/WT) cells. Tubulin was the loading control of protein samples. (**C**) *In vitro* proliferation of Du145/WT, Du145/Ctrli #1, Du145/Cyr61i #1, and Du145/Cyr61i-Cyr61SM cells was examined by MTT assay. (**D**) Crystal violet assay of colony formation of Du145/Cyr61i #1 and Du145/Ctrli #1 cells. Cells (3 × 10^3^) were seeded each well in 12-well plates and photographed 2 weeks later. Number of cells was measured by detecting under OD 600 nm. A typical experiment was shown. (**E**) Crystal violet assay of PC3/Ctrl, PC3/Cyr61i, PC3/Cyr61i-Cyr61SM and PC3/Cyr61 cells was taken under the same way. (**F**) Du145 cells were infected with lentivirus expressing both EGFP and control RNAi or Cyr61 RNAi as evidenced by representative light-field or fluorescence photos. (**G**) LnCap cells were infected with lentivirus either expressing Cyr61 (LnCap/Cyr61) or the control GFP (LnCap/GFP). *In vitro* proliferation of LnCap/Cyr61 and LnCap/GFP cells was examined by MTT assay. (**H**) Schematic diagram showing the domain structure of wild-type Cyr61, silent mutation of Cyr61. Six nucleic acid changed Cyr61SM without changing the amino acid of Cyr61 making it resistant to the RNA interference function of 1# siRNA sequence. ^*^P<0.05, ^**^P<0.01, Student's *t*-test.

To knock down the endogenous Cyr61, we selected three siRNA hairpin sequences against different sites of Cyr61 mRNA that were designed using Ambin web software: (1) GGCCAGAAATGTATTGTTC; (2) GAAATGCAGCAAGACCAAG; and (3) GAACGTCATGATGATCCAG. These sequences were cloned to the FG12 vector and RNA interference (RNAi) cell lines were produced as described previously (Qin *et al*, 2003). A silent mutant Cyr61 expression construct was made resistant to an siRNA sequence. Between the nucleic acids 676 and 694, GGCCAGAAATGTATTGTTC were replaced with GGACAAAAGTGCATCGTAC. The silent mutant Cyr61 was delivered into the Cyr61-silenced Du145 cells with lentivirus (Figure 1H).

### Forced expression of Cyr61 in Du145 and LnCap cells

A modified lentivirus-based FG12 vector with a CMV promoter to introduce Cyr61 gene was expressed in Du145 and LnCap cells (Qin *et al*, 2003). After infection, the cells were sorted by FACS to collect GFP-positive cells.

### Western blot analysis

Total cell lysates (40 *μ*g of protein) were separated by 10% SDS–PAGE gels and electrotransferred onto polyvinylidene difluoride membranes (Millipore, Bedford, MA, USA). After blocking with 5% milk, the membranes were probed with primary antibodies at 1 : 1000 dilutions. The membrane was washed and then incubated with horseradish peroxidase-conjugated secondary antibodies for 1 h. Immunoreactive proteins were detected with enhanced chemiluminescence reagents (Pierce, Rockford, IL, USA) and photographed with Kodak X-Omat blue autoradiography film.

### Cell proliferation analysis

To analyse the effect of Cyr61 on PCa cell proliferation activity, MTT assay and crystal violet assay were taken. In MTT assay, cells were plated into 96-well plates at 2 × 10^3^ cells per well, cultured in 0.5∼1% FBS RPMI-1640 for various durations, and cell numbers were measured by MTT assay according to the protocol provided by MTT manufacturer. In crystal violet assay, equal number of cells and their control cells were seeded in 12-well plates and cultured in media supplemented with 0.5∼1% FBS for 7 days; media were changed every other day. Cellular growth was stopped after 7 days in culture by removing the media and adding 0.5% crystal violet solution in 20% methanol. After staining for 5 min, the fixed cells were washed with phosphate-buffered saline (PBS) and photographed (Thomas *et al*, 2004).

### Soft agar assay

Cells were plated in 24-well flat-bottomed plates using a two-layer soft agar system with 1 × 10^3^ cells per well in a volume of 400 *μ*l per well as described earlier (Munker *et al*, 1986). After 14 days of incubation, colonies were counted and measured. All the experiments were repeated at least three times using triplicate plates per experimental point.

### Purification of recombinant hCyr61 protein

Cyr61 was cloned into PIZT/V5-His vector between *Eco*RI and *Xba*I, followed by transfecting into SF9 insect cells with Cellfectin (Invitrogen). Cells were selected for antibiotic resistance to Zeocin. A Zeocin-resistant pool of cells stably expressed secreted r-hCyr61 recombinant protein. Cyr61-contained medium was collected and the recombinant protein was purified with chromatography on Ni^2+^ metal-chelating resins.

### Tumorigenesis assay *in vivo*

Stably infected and FACS-sorted cells (1 × 10^7^ cells per flank) suspended in 200 *μ*l of RPMI and 150 *μ*l Matrigel were injected into 5-week-old female SCID mice (CB-17TM/IcrCrl-scidBR) purchased from Shanghai Laboratory Animal Center, CAS (Shanghai, China) and treated in accordance with the American Association for the Accreditation of Laboratory Animal Care guidelines. Each animal was subcutaneously injected at two sites in the flanks. The resulting tumours were measured once a week and tumour volume (mm^3^) was calculated using the standard formula: length × width × height × 0.5236. Tumours were harvested 8 weeks after injection and individually weighed.

### Wound-healing assays

Cells were seeded onto six-well dishes at 1 × 10^5^ per well in growth medium. Confluent monolayers were starved overnight in assay medium and a single scratch wound was created using a micropipette tip. Cells were washed with PBS to remove cell debris, supplemented with assay medium, and monitored. Images were captured by phase microscopy using a × 10 objective at 0 and 24 h post-wounding. The percentage of cells in the wound-healing area was averagely calculated from four experiments.

### Boyden chamber assay

Boyden chamber (8-*μ*m pore size polycarbonate membrane) was obtained from Neuroprobe Corp., Bethesda, MD, USA. Cells (2 × 10^5^) in 0.05 ml medium with 1% FBS were placed in the upper chamber, and the lower chamber was loaded with 0.152 ml medium containing 10% FBS. Cells that migrated to the lower surface of filters were detected with traditional H&E staining, and five fields of each well were counted after 4–24 h of incubation at 37°C with 5% CO_2_. Three wells were examined for each condition and cell type, and the experiments were repeated thrice. For inhibitor experiment, the migrated cells were detected as described above only after overnight incubation with or without series concentrations of NSC23766.

### Cloning and production of GST-PAK-CD fusion protein

The Rac1 activity assay was based on the Rap1 activity assay described by Franke *et al* (1997). We used a glutathione-*S*-transferase (GST)-PAK-CD (PAK-CRIB domain) fusion protein, containing the Rac1- and Cdc42-binding regions from human PAK1B (GenBank accession number AF071884). A fragment encoding amino acids 56–272 of PAK1B was generated by standard PCR using the oligos 5′-AGCTGGATCCATTTTACCTGGAGAT-3′ and 5′-AGCTCTCGAGATTTCTGGCTGTTGGATGTC-3′, digested with *Bam*HI/*Xho*I, and then inserted between *Bam*HI with *Xho*I sites of pGEX4T1 (Pharmacia Biotech, Piscataway, NJ, USA) to yield GST-PAK-CD. *Escherichia coli* BL21 cells transformed with the GST-PAK-CD construct were grown at 37°C to an absorbance of 0.3. Expression of recombinant protein was induced by the addition of 0.1 mM isopropylthiogalactoside for 2 h. Cells were harvested, resuspended in lysis buffer (50 mM Tris pH 8, 2 mM MgCl_2_, 0.2 mM Na_2_S_2_O, 10% glycerol, 20% sucrose, 2 mM dithiothreitol, 1 *μ*g ml^−1^ leupeptin, 1 *μ*g ml^−1^ pepstatin, and 1 *μ*g ml^−1^ aprotinin), and then sonicated. Cell lysates were centrifuged at 4°C for 20 min at 45 000 **g** and the supernatant was incubated with glutathione-coupled Sepharose 4B beads (Pharmacia Biotech) for 30 min at 4°C. Protein bound to the beads was washed three times in lysis buffer and the amount of bound fusion protein was estimated using Coomassie-stained SDS gels.

### Rac1-GTP pull-down assay

The Rac1 activity assay was performed by pull-down using GST fusion with the protein-binding domain of p21-activated kinase (GST-CIRB). Briefly, Du145 cells were plated onto the plate at a density of approximately 2 × 10^6^ cells per 10 cm dish. When grown to 60–70% confluence, cells were washed twice with PBS, then lysed for 5 min in 250 *μ*l ice-cold eukaryotic lysis buffer (25 mM Tris (pH 7.4) 1 mM EDTA, 5 mM MgCl_2_, 1 mM DTT, 0.1 mM EGTA, 100 mM NaCl, 1% NP-40, 5% glycerol, 1 mM PMSF, 1 *μ*g ml^−1^ aprotinin, 1 *μ*g ml^−1^ leupeptin and 1 *μ*g ml^−1^ pestatin) and then incubated for 30 min with GST-PAK fusion protein that had been adsorbed to glutathione agarose beads. Beads were washed three times with 500 *μ*l of cold eukaryotic lysis buffer and then resuspended in 10 *μ*l reducing electrophoresis sample buffer (2% SDS, 10% glycerol, 80 mM (Tris pH 6.8), 2 mM EDTA, 100 mM DTT, and 0.1% bromophenol blue) and analysed by SDS–PAGE in a 12% gel. Following electrophoresis, samples were transferred to a nitrocellulose membrane and immunoblotted with mouse anti-human Rac1 antibody at a dilution of 1 : 1000. Normalised Rac1 activity values were determined by dividing the amount of Rac1 in the pull-down by the amount of Rac1 in the loading control.

### *In vivo* bioluminescence imaging of tumour cells

Du145 cells were transfected with plasmids expressing firefly luciferase (PGL4-CMV-Luc2; Longmed Corporation, Beijing, China) using Lipofectamine 2000. Cells were selected for antibiotic resistance with G418. The surviving colonies were screened for bioluminescence in complete media supplemented with 150 *μ*g ml^−1^ D-luciferin by *in vitro* imaging using the IVIS_ Imaging System (Xenogen, Alameda, CA, USA). Bioluminescent antibiotic-resistant clones were amplified in culture and characterised for stable luminescence *in vitro* and tumorigenic potential *in vivo*. One positive cell line, Du145-luc2-7, was selected for further studies. Du145-luc2-7 cells were infected with control siRNA virus (Du145-Luc2/Ctrli), Cyr61 siRNA virus (Du145-Luc2/Cyr61i), null virus (Du145-Luc2/GFP), and Cyr61 expression virus (Du145-Luc2/Cyr61), respectively. Four pools of Du145-Luc2/Ctrli, Du145-Luc2/Cyr61i, Du145-Luc2/GFP, and Du145-Luc2/Cyr61 were acquired after FACS sorting. They were injected, respectively, into the nude mice from the tail vein. Mice were anaesthetised by inhalation of 3% isoflurane/oxygen mixture delivered by the Xenogen XGI-85-port gas anaesthesia system. Imaging and image analysis were performed following the Xenogen protocol using a cooled CCD camera system (IVIS-100; Xenogen) and LivingImage software (Xenogen). The photographs were taken weekly.

### Immunohistochemistry

For immunohistochemistry, primary PCa samples and their corresponding normal tissues were frozen in a cryostat chamber, and 10 *μ*m sections were collected on glass slides. The sections were fixed in ice-cold acetone for 30 min, washed in 0.01 M PBS (8 mM Na_2_HPO_4_, 2 mM NaH_2_PO_4_, and 150 mM NaCl) for 3 × 5 min, blocked for 1 h in 0.01 M PBS supplemented with 0.3% Triton X-100 and 5% normal goat serum, and then incubated with Cyr61 antibody (1 : 500) at 4°C overnight. After brief washes in 0.01 M PBS, sections were incubated for 2 h in 0.01 M PBS with horseradish peroxidase-conjugated goat anti-rabbit IgG (1 : 1000), followed by development with 0.003% H_2_O_2_ and 0.03% DAB in 0.05 M Tris-HCl (pH 7.6). Immunohistochemistry for each sample was performed at least three times, and all sections were counterstained with haematoxylin.

### Statistical analysis

The results were representative of at least three independent experiments performed in triplicate and were expressed as the mean±s.d. Statistical analysis of the data was performed using Student's *t*-test.

## Results

### Cyr61 stimulated the growth of PCa cells *in vitro*

We first examined the expression of Cyr61 in the four PCa cell lines 22RV1, LnCap, Du145, and PC3. In Figure 1A, the two highly malignant cell lines – PC3 and Du145 – exhibited significantly higher Cyr61 expression, whereas Cyr61 was nearly undetectable in the other two less malignant cell lines – LnCap and 22RV1 (Figure 1A). The level of Cyr61 in Du145 cells was fairly moderate among the four PCa cell lines; therefore, we explored the function of Cyr61 in Du145 cells by siRNA and antibody neutralisation, as well as forced expression and protein treatment, whereas overexpression and r-hCyr61 protein stimulation were employed in the study on LnCap cells.

After successful knockdown of endogeneous Cyr61 by RNAi (Figure 1B), we examined the cell growth using MTT assay, and the result showed that downregulation of Cyr61 in DU145 cells resulted in an obvious decrease in proliferation. Consistent with this, the introduction of the silent mutant Cyr61 rescued Cyr61 expression and the proliferation of Du145 cells (Figure 1C and H). Crystal violet experiment showed that the clones derived from Du145/Cyr61i cells were much smaller and fewer than those formed by Du145/Ctrli cells; in contrast, elevated Cyr61 level stimulated Du145 cells to form larger and more clones (Figure 1D). Similar results could also be observed in PC3 cells (Figure 1E). These results suggested that downregulation of Cyr61 Du145 cell proliferation and forced expression of Cyr61 in LnCap cells promoted cell proliferation (Figure 1G). Therefore, Cyr61 was required for the proliferation of PCa cells, which were consistent with their function reported in benign prostatic hyperplasia (Sakamoto *et al*, 2004).

### Cyr61 promoted the tumorigenicity of Du145 cells both *in vitro* and *in vivo*

**Figure 2:**
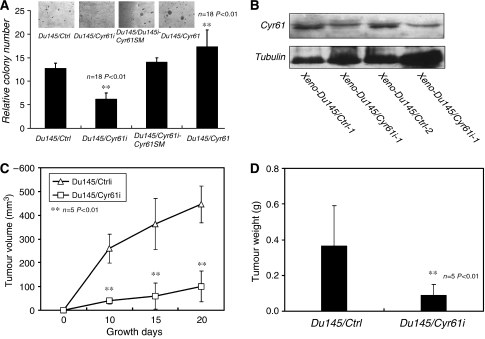
Silencing of Cyr61 suppressed the tumorigenicity of Du145 cells. (**A**) Soft agar assay showed that Du145/Cyr61i #1 cells formed much fewer colonies in comparison with Du145/Ctrli #1 cells. Rescued expression of Cyr61 with cyr61 silent mutation and forced expression of cyr61 with lentivirus form much more and larger compared with the Cyr61/Cyr61i. (**B**) Xenografts were taken out after the mice were killed. Cyr61 expression level was detected by western blot. (**C**) Du145/Cyr61i #1 or Du145/Ctrli #1 cells (1 × 10^7^) were subcutaneously injected into the dorsal skin of nude mice. Tumour volumes were measured every 7 days, and each point represented the mean volume±s.d. from five independent experiments. (**D**) Four weeks later, the mice were killed and the tumours were picked up for weighing. Each histogram represented the mean weight±s.d. from five independent experiments. ^**^P<0.01, Student's *t*-test.

As the knockdown of Cyr61 inhibited the proliferation of PCa cells, we wondered whether it would affect the tumorigenicity either. Soft agar test was carried out to examine the anchorage-independent growth, which is a typical characteristic of the tumorigenicity of cancer cells *in vitro* (Dodson *et al*, 1981; Grisham *et al*, 1991). Du145/Ctrli, Du145/Cyr61i, Du145/Cyr61i-Cyr61SM, and Du145/Cyr61 cells were inoculated on the upper layer of soft agar. After 2 weeks, a number of obvious colonies of Du145/Ctrli cells were observed in each well, whereas much fewer colonies were formed by Du145/Cyr61i cells. Rescuing Cyr61 expression in Du145/Cyr61i cells by byCyr61SM resulted in almost as many colonies as those formed by Du145/Ctrli cells. Furthermore, the colonies derived from Du145/Cyr61i-Cyr61SM cells were larger than those from Du145/Cyr61i cells. Forced expression of Cyr61 in Du145 cells produced more and larger colonies when compared with the Du145/Ctrli cells (Figure 2A). These results indicated that Cyr61 promoted the tumorigenicity of Du145 cells *in vitro*.

To further examine the effect of Cyr61 on the tumorigenicity of Du145 cells *in vivo*, either Du145/Cyr61i or Du145/Ctrli cells were subcutaneously injected into nude mice. Tumour volumes were measured every 7 days, and the result showed that Du145/Cyr61i cells formed much smaller tumours when compared with Du145/Ctrli cells (Figure 2C). Four weeks later, the mice were killed and all the tumours were weighed. In accordance with their volumes, the weight of tumours from Du145/Cyr61i cells was lighter than those derived from Du145/Ctrli cells (Figure 2D). Proteins from the xenografts were extracted and the expression of Cyr61 was examined. As shown in Figure 2B, the level of Cyr61 in the Du145/Cyr61i xenografts was lower than that in the Du145/Ctrli xenografts. These data thus suggested that Cyr61 downregulation also impaired the tumorigenicity of Du145 cells *in vivo*.

### Cyr61 was essential for the migration of PCa cells *in vitro*

**Figure 3:**
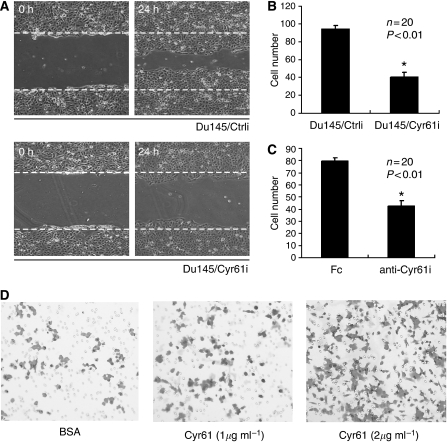
Silencing of Cyr61 inhibited the migration of Du145 cells. (**A**) Wound-healing assay for either Du145/Ctrli #1 or Du145/Cyr61i #1 cells. (**B**) Boyden chamber assay for Du145/Ctrli #1 or Du145/Cyr61i #1 cells, and the cells migrated through the polycarbonate membrane were counted following H&E staining. Student's *t*-test (^*^P<0.01). (**C**) Boyden chamber assay for Du145 cells treated with either rabbit IgG (Fc) or the antibody against Cyr61 (anti-Cyr61) and the cells migrated through the polycarbonate membrane were counted following H&E staining. Student's *t*-test (^*^P<0.01). (**D**) Boyden chamber assay for LnCap cells treated with either BSA (1 *μ*g ml^−1^) or the purified Cyr61-his protein. Cyr61 stimulates LnCap cell migration in a dosage-dependent manner.

A number of studies have identified that Cyr61 functioned as a ligand of integrins (Kireeva *et al*, 1998) that play pivotal roles in cell migration (Hood and Cheresh, 2002; Carragher and Frame, 2004; Juliano *et al*, 2004; Moschos *et al*, 2007). Thus, we investigated the effects of Cyr61 on the mobility of Du145 and LnCap cells, which bore different levels of Cyr61 expression, respectively. Wound-healing results demonstrated that much fewer Du145/Cyr61i cells moved into the wound area than the Du145/Ctrli cells in the same interval (Figure 3A). Similarly, Boyden chamber assay revealed that the migration ability was impaired in Du145/Cyr61i cells compared with Du145/Ctrli cells (Figure 3B). To further confirm the effect of Cyr61 on cell migration, we used antibodies against Cyr61 (SC-13100) to block the endogenous Cyr61 in Du145 cells. Consistent with the results of an earlier study, the Boyden chamber assay showed that the migration was markedly suppressed upon antibody blocking (Figure 3C). As Cyr61 is a secreted protein, we hypothesised that Cyr61 exerted its effect on migration in a paracrine manner. To examine this hypothesis, we purified r-hCyr61 protein from insect cells and treated the LnCap cells, which express very low levels of Cyr61, with the r-hCyr61 protein. Expectedly, LinCap cells showed elevated migration ability in a dosage-dependent manner in r-hCyr61 protein treatment (Figure 3D). Thus, these results suggested that Cyr61 was closely involved in the migration of PCa cells.

### Downregulation of Cyr61 increased focal adhesion assembly in Du145 cells

**Figure 4:**
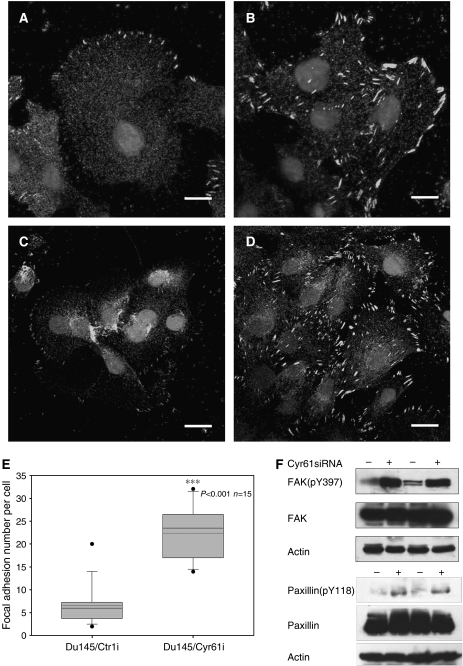
Silencing of Cyr61 changed the intensity and distribution of focal adhesion. Immunofluorescence of FAK and paxillin, two markers of focal adhesion, on either Du145/Ctrli #1 ((**A**) for FAK staining and (**C**) for paxillin staining) or Du145/Cyr61i #1 cells ((**B**) for FAK staining and (**D**) for paxillin staining). Scale Bar=10 *μ*m. (**E**) Increased focal adhesion formation upon reduced Cyr61 expression. Box-and-whisker plots of paxillin-positive-stained points within Du145/Ctrli or Du145/Cyr61i plated on FN for 8 h (^***^P<0.001; *n*=15 cells per point). (**F**) Increased FAK (pY397) and paxillin (pY118) levels upon reduced Cyr61 expression. Western blot detected protein level of total FAK and paxillin, together with FAK (pY397) and paxillin (pY118). *β*-Actin was used as a loading control.

As the effects of integrins on migration often involve the regulation of focal adhesion (FA) (Miyamoto *et al*, 1995; Burridge and Chrzanowska-Wodnicka, 1996), we examined the status of FA after downregulation of Cyr61 by immunofluorescence of FAK (Figure 4A and B) and paxillin (Figure 4C and D), both of which serve as markers of FA (Miyamoto *et al*, 1995; Burridge and Chrzanowska-Wodnicka, 1996). As shown in Figure 4E, Du145/Cyr61i cells formed more FAs than Du145/Ctrli cells. Box-and-whisker plots of paxillin-positive-stained points within Du145/Ctrli or Du145/Cyr61i plated on FN for 8 h are shown. In Du145/Ctrli cells, the staining of FAK (Figure 4A) and paxillin (Figure 4C) was weak and mainly appeared on the polarised edge. In contrast, in Du145/Cyr61i cells, the staining signals of (Figure 4B) both FAK and paxillin (Figure 4D) were much more intense and exhibited uniform membrane distribution. This difference indicated altered FA assembly (Miyamoto *et al*, 1995; Burridge and Chrzanowska-Wodnicka, 1996). Phosphorylated FAK or paxillin, which are activated forms of the molecule, was probed with anti-FAK (pY397) or anti-paxillin (pY118) monoclonal antibodies, respectively. The phosphorylation level of both FAK and paxillin was elevated upon silencing of Cyr61 (Figure 3F), which indicated the increased activation of the two molecules. As the increase of FA often promotes the adhesion of host cells with the extracellular matrix and the loss of polar distribution of FA inhibits cell mobility (Hood and Cheresh, 2002; Carragher and Frame, 2004), the alteration of FA assembly in Du145/Cyr61i cells was consistent with their impaired mobility.

### Effect of Cyr61 on PCa cell migration was mediated through the activity of Rac1

**Figure 5:**
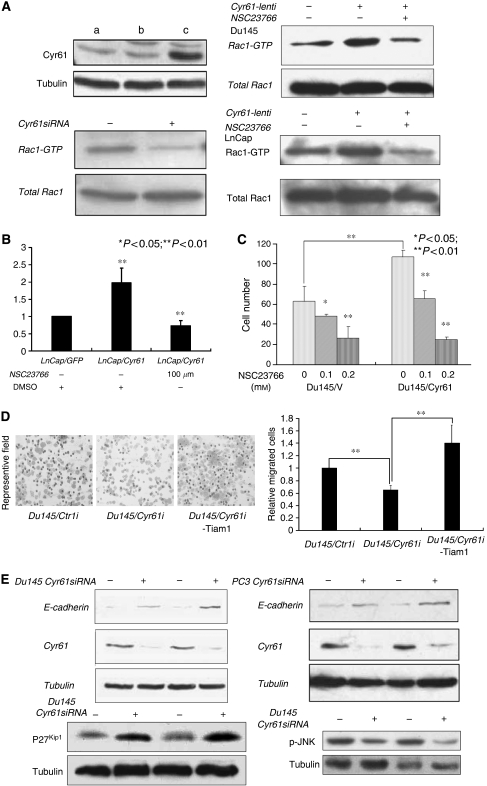
Rac1 was implicated in the regulation of Du145 cell migration by Cyr61. (**A**) Protein levels of Cyr61 following forced expression of Cyr61 in Du145 cells. Lane a: Du145/WT cells; lane b: Du145 cells infected with lentivirus expressing GFP (Du145/GPF); lane c: Du145 cells infected with Cyr61 expression lentivirus (Du145/Cyr61). Activity of Rac1 was detected following silencing (down left) or forced expression of Cyr61 in Du145 cells (up right) and LnCap cells (down right) or treated with Rac1-specific inhibitor NSC23766. (**B**) Boyden chamber assay for either LnCap/GPF or LnCap/Cyr61 cells with or without priorly treated with NSC23766. ^**^P<0.01. (**C**) Boyden chamber assay for either Du145/V or Du145/Cyr61 cells priorly treated with a series of concentrations of Rac1-specific inhibitor NSC23766. ^*^P<0.05; ^**^P<0.01. (**D**) Boyden chamber assay for either Du146/Ctrli or Du145/Cyr61i or Du145/Cyr61 transfected with Rac1 stimulator Tiam1. ^*^P<0.01. (**E**) Rac1 downstream molecules that can affect cell migration and proliferation were detected both in Du145 cells and PC3 cells after silencing of Cyr61 expression. E-cadherin and p27^Kip1^ protein level are elevated after silencing of Cyr61. However, protein levels of phosphorylated JNK (p-JNK) were decreased following silencing of Cyr61 expression in Du145 cells with two different siRNA sequences.

To address the molecular mechanisms by which Cyr61 regulated the migration of Du145 cells, we further examined some key molecules involved in the integrin signalling pathways, such as ERK, AKT, and JNK (Juliano *et al*, 2004). In Du145/Cyr61i and the Du145/Ctrli cells, the activating forms of both ERK and AKT were almost the same (data not shown), whereas the activated JNK markedly decreased in the Du145/Cyr61i cells (Figure 5E). Previous studies have proved that the small GTP-binding protein Rac1 played a key role in the phosphorylation of JNK as well as cell migration (Juliano *et al*, 2004). Therefore, we assumed that the loss of activated JNK might be induced by a decreased level of activated Rac1. To test our hypothesis, the activated Rac1 was pulled down with GST-PKA (see Materials and Methods), and the result showed that there was much less activated Rac1 in Du145/Cyr61i cells than in Du145/Ctrli cells (Figure 5A). For further studying the molecular mechanism, we compared the activation of Rac1 in LnCap and Du145 cells overexpressing Cyr61 (LnCap/Cyr61 and Du145/Cyr61) with that in their parental cells. As expected, Cyr61 overexpression indeed enhanced the activation of Rac1 (Figure 5A) as well as the migration in both LnCap and Du145 cells (Figure 5B and C). To confirm that the influence of Cyr61 on the migration of PCa cell was Rac1 dependent, we employed a specific inhibitor NSC23766 for Rac1 to manipulate the activity of endogeneous Rac1 in LnCap/Cyr61 and Du145/Cyr61 cells. As shown in Figure 5B and C, the migration could be inhibited by NSC23766 in both LnCap and Du145 cells (Figure 5B and C). To further confirm the effect of Rac1 on Cyr61-induced cell migration, we transfected the Du145/Cyr61i cells with T-cell lymphoma invasion and metastasis 1 (Tiam1), which was reported as a specific activator of Rac1 (Hamelers *et al*, 2005; Cruz-Monserrate and O’Connor, 2008). Tiam1-transfected Du145/Cyr61i cells effectively reversed the migration-suppression effects of Cyr61 silencing (Figure 5D).

Besides JNK, we also looked into other molecules downstream of Rac1, which are involved in both cell proliferation and migration. Protein level of E-cadherin was elevated in Cyr61-silenced Du145 and PC3 cells. p27^kips^, a famous CDK suppressor that was recently reported to have an important suppression function in cell migration (Bremnes *et al*, 2002; Supriatno *et al*, 2003), was also upregulated during silencing of Cyr61 in Du145 cells (Figure 5E). Therefore, the effects of Cyr61 on migration of Du145 cells might be mainly mediated through Rac1 signalling.

### Cyr61 stimulates Du145 metastasis *in vivo* and Cyr61 expression profile in clinical samples

**Figure 6:**
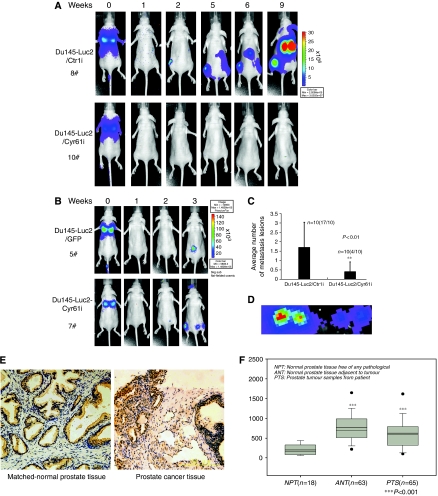
Silencing of Cyr61 eliminated the systemic tumour growth of Du145 cells *in vivo*. (**A**, **B**, **C,** and **D**) Either Du145-Luc2/Ctrli #1 or Du145-Luc2/Cyr61i #1 cells (1 × 10^7^) and 5 × 10^6^ of either Du145-Luc2/GFP or Du145-Luc2/Cyr61 cells were injected into each 10 mice tail vein separately. The bioluminescence images were acquired using the IVIS imaging box at indicated time points. Representative mice (8# and 10#) of Du145-Luc2/Ctrli #1, Du145-Luc2/Cyr61i #1 (**A**) and Du145-Luc2/GFP, Du145-Luc2/Cyr61 (**B**) were presented for each time point. (**C**) Average number of metastasis lesions was qualified. (**D**) The metastasis lesion was picked out to confirm that bioluminescence signalling is not artificial. (**E**) Immunohistochemistry of Cyr61 in clinical prostate cancer samples and matched normal prostate tissue. Scale bar=100 *μ*m. (**F**) Elevation of Cyr61 level upon tissue adjacent to tumour and the tumour samples compared with normal prostate tissues free of any pathological alteration; box-and-whisker plots of Cyr61 expression level at different stages of prostate cancer tissues. ^***^P<0.001; ^**^P<0.01.

Considering the effect of Cyr61 on cell migration, we suspect it might also influence the metastasis of PCa cells *in vivo*. First, we constructed Du145-Luc2 cells, which were stably transfected with a firefly luciferase gene (see Materials and Methods), and subsequently infected with lentivirus delivering Cyr61 siRNA (Du145-Luc2/Cyr61i), control siRNA (Du145-Luc2/Ctrli), null (GFP only), or Cyr61, respectively. Four pools enriched with Du145-Luc2/Cyr61i, Du145-Luc2/Ctrli, Du145-Luc2/GFP, and Du145-Luc2/Cyr61 cells were selected by FACS. Also, cells from each pool were injected into the mouse through tail vein. It is well known that an important procedure of tumour metastasis *in vivo* is the formation of new colonies of tumour cells after movement in blood vascular system (Steeg, 2006), and the experimental model we used mimicked this process. As shown in Figure 6A, Du145-Luc2/Ctrli cells formed more tumour foci, which could be detected by bioluminescence imaging after a relatively short latency; whereas Du145-Luc/Cyr61i cells produced no tumour foci in the same time period. The average metastasis lesions were quantitated (Figure 6C). To exclude the possibility that the signal examined might be artificial, after the mouse were killed, we found out the metastasis lesions according to the previously obtained data (Figure 6D). Student's *t*-test revealed statistically significant difference between the control and Cyr61-silenced groups (*P*<0.01). Similarly, the Du145-Luc2/Cyr61 cells led to much more metastasis lesions than Du145-Luc2/GFP cells (Figure 6B). Conclusively, the results indicated the crucial role of Cyr61 in the metastasis of Du145 cells *in vivo*.

### Cyr61 expression was changed with PCa progression

After analysing the Cyr61 expression profile in the gene expression array-based database of human metastatic prostate tumours and primary prostate tumours (GSE6919), we found that the normal prostate tissues showed very low level of Cyr61 expression, whereas the tumour-adjacent tissues and the tumour tissues demonstrated a significantly higher expression level of Cyr61 (*P*<0.001, Figure 6F). However, the expression level of Cyr61 in metastatic prostate tumour samples decreased when compared with that in the early stage of PCa (Figure 6F).

Metastasis largely results from the interaction between tumour cells and the surrounding environment. Yang *et al* (2005) found that mesenchymal stem cells within tumour stroma promoted breast cancer metastasis through the secretion of the chemokine CCL5. It was reported that the stromal expression of IGFBP3 was important for PCa progression (Massoner *et al*, 2008). Recently, Karnoub *et al* (2007) reported that stromal expression of CTGF promoted angiogenesis and tumorigenesis of PCa. These findings indicated the potential function of stromal Cyr61 in metastasis and tumour progression. Consistent with these studies, we found that Cyr61 indeed existed in the fibroblast cells in the region of cancerous tissues (Figure 6E). We further analysed the expression of Cyr61 in 20 pairs of clinical malignant PCa samples and their matched normal prostate tissues by immunostaining. A representative result showed that the expression of Cyr61 was mild in both epithelial cells and stromal fibroblast cells in normal prostate tissues, whereas higher level of Cyr61 expression was observed in epithelial PCa foci and the stromal fibroblast cells in tumour samples (Figure 6E), which indicated that Cyr61 played a promotive role in the tumorigenesis of PCa and served as an important paracrine growth factor to prompt the metastasis of malignant PCa cells.

## Discussion

In this study, we explored the functions of Cyr61 in several representative PCa cell lines, including LnCap, Du145, and PC3 cells, and found that Cyr61 facilitated the proliferation and migration of the tumour cells, suggesting that Cyr61 might act as an oncoprotein in PCas.

Cyr61 is a well-established ligand for several integrins (Kireeva *et al*, 1998), including *α*v*β*3 and *α*6*β*1, which were expressed in Du145 cells (Witkowski *et al*, 1993). Responding to the alteration of extracellular environment, for example, in ligand engagement, integrins can elicit intracellular signal pathways to regulate cell survival, proliferation, gene transcription, adhesion to ECM, and migration (Hood and Cheresh, 2002; Carragher and Frame, 2004; Juliano *et al*, 2004; Moschos *et al*, 2007). Earlier studies have demonstrated that Cyr61 induced cell proliferation, adhesion, and angiogenesis through activation of integrin (*á*v*â*3) in endothelial cells (Babic *et al*, 1998). It was possible that the effect of Cyr61 exerted in Du145 cells might also be mediated through integrins.

In fact, we observed that the loss of Cyr61 facilitated the assembly of FAs, which is the intermediate for the interaction between cytoskeleton and ECM, and is largely regulated by integrin signalling pathways (Miyamoto *et al*, 1995; Burridge and Chrzanowska-Wodnicka, 1996; Juliano *et al*, 2004). Integrin-induced assembly or disassembly of FAs often resulted in the enhancement or attenuation of cell adhesion to the ECM, respectively, and the dynamic regulation of FA was a crucial determinant in cell migration (Hood and Cheresh, 2002; Carragher and Frame, 2004). The recruitment of many factors including FAK and paxillin promoted the assembly of FA, thus stabilising the interaction between cytoskeleton and ECM, which enhanced the cell adhesion while inhibited cell migration (Hood and Cheresh, 2002; Carragher and Frame, 2004). In contrast, the degradation or separation of FA components led to the disassembly of FA and elevated cell motility (Hood and Cheresh, 2002; Carragher and Frame, 2004). In our study, we found that knockdown of Cyr61 resulted in an excessive assembly of FAs in Du145 cells, which was consistent with the inhibition of migration. These results also reflected that loss of Cyr61 in Du145 cells might interfere with the normal regulation of FAs by integrins. It has been reported that CTGF promoted mesangial cell migration by disassembling FAs (Crean *et al*, 2004). As a high similarity existed in the structure and function between Cyr61 and CTGF, and both of them could serve as ligands for integrins (Brigstock, 2003), it was not strange that Cyr61 regulated FA in a similar way as CTGF does.

Our results also showed that the activation of Rac1, one important effector downstream of integrins (Hood and Cheresh, 2002; Carragher and Frame, 2004), was dramatically inhibited by the knockdown of Cyr61. The suppressive effect on cell migration by Cyr61 knockdown was also largely due to the decreased level of activated Rac1, which has also been shown important in cell migration in some other kinds of cancer cells (Almeida *et al*, 2000; Juliano *et al*, 2004). Rac1 is a member of Rho family and regulates cell migration by stimulating actin polymerisation to form lamellipodia (Carragher and Frame, 2004). As the extension of lamellipodia is an important step for cell migration, the decrease of mobile lamellipodia, which resulted from the inactivation of Rac1, inevitably lowers the cell motility. Therefore, Cyr61 might regulate the activity of Rac1 through the integrin pathway and then affect the migration of PCa cells. So far, several pathways have been found to participate in the regulation of migration by integrins, including AKT, ERK, and JNK signalling (Almeida *et al*, 2000; Juliano *et al*, 2004). However, we only detected the change in the activated status of JNK, suggesting that Cyr61 could activate JNK through integrin. Moreover, JNK is a downstream target of integrin-related Rac1 (Almeida *et al*, 2000; Juliano *et al*, 2004). Therefore, our results revealed that a novel Cyr61-integrin-Rac1-JNK signalling regulated the migration of malignant PCa cells.

Our *in vivo* study further showed that the metastasis of Du145 was inhibited by the silencing of Cyr61. A microarray analysis was conducted (Bacac *et al*, 2006) (data accessible at NCBI GEO database, accession GSE5945) to evaluate the response of stromal cells to tumour invasion. Cyr61 expression level was found higher in the stromal cells from invasive prostate tumour tissue than that in the stromal cells from intraepithelial neoplasias, suggesting the important role of Cyr61 in the metastasis of malignant PCa cells. Metastasis is the major barrier to eradicate cancers as well as the primary cause of death in patients (Steeg, 2006). So far, a lot of research have explored the complicated processes of cancer metastases and uncovered the general process of metastases as follows: tumour cells that have already acquired weakened adhesion and enhanced migration ability first separate from neighbouring cells; then the separated tumour cells enter into the circulation system (lymph or blood); during the process of transporting in the circulation system, the tumour cells translocated in the new proper tissues or organs by invasion and form new tumour foci, from which metastatic cancers eventually develop (Steeg, 2006). During the process, the abilities to propagate, migrate, and invade the metastatic tumour cells were all dramatically increased compared with homologous normal cells and non-metastatic tumour cells (Steeg, 2006). In our experimental model of metastasis, the tumour cells were directly injected into the blood system, which mimicked the movement of tumour cells in the circulation system. We found that Cyr61-downregulated Du145 cells could not develop metastatic lesions *in vivo*, whereas Du145 cells with normal Cy61 expression easily formed new tumour foci. The difference might be due to the decrease in cell proliferation and/or the attenuation of cell migration ability, which was proved in our study. In addition, Cyr61 was found to induce matrix metalloproteinase-1 production, which promoted the invasion of breast cancers (Nguyen *et al*, 2006). Moreover, Cyr61 was also a tumour-promoting protein in breast cancers (Liotta and Kohn, 2001; Xie *et al*, 2001a), which was similar to our findings in PCas; thus the loss of Cyr61 in PCa cells might also inhibit cell invasion by blocking the expression of matrix metalloproteinase-1. Recently, Cyr61 expression was also found to promote metastasis of human pancreatic cancer (Holloway *et al*, 2005). Therefore, Cyr61 might be a useful marker for metastasis in several types of malignant cancers including PCas.

Taken together, our results herein displayed that Cyr61 plays an important role in the proliferation and migration of malignant PCa cells, and the latter was largely due to the regulation of Cyr61 on Rac1 activity, as well as related FA assembly. The *in vivo* study further demonstrated that Cyr61 was also involved in the metastasis of PCa. Our results seemed contrary to the earlier report that Cyr61 might act as a tumour suppressor in PCa (Pilarsky *et al*, 1998). In that study, the researchers mainly used low-stage or well-differentiated PCa samples (Pilarsky *et al*, 1998). Therefore, we speculated that Cyr61 might play distinct roles in PCas with different degrees of malignance; however, this hypothesis required further investigations. After all, we successfully inhibited tumorigenicity of PCa cells both *in vitro* and *in vivo* by downregulating Cyr61 expression, and this suggested a potential therapeutic strategy for malignant PCas.
